# Longitudinal associations of sociodemographic, lifestyle, and clinical factors with alcohol consumption in colorectal cancer survivors up to 2 years post-diagnosis

**DOI:** 10.1007/s00520-021-06104-0

**Published:** 2021-03-24

**Authors:** Dóra Révész, Martijn J. L. Bours, Johannes A. Wegdam, Eric T. P. Keulen, Stéphanie O. Breukink, Gerrit D. Slooter, F. Jeroen Vogelaar, Matty P. Weijenberg, Floortje Mols

**Affiliations:** 1 Center of Research on Psychological and Somatic disorders (CoRPS), Department of Medical and Clinical Psychology, Tilburg University, PO Box 90153, 5000 LE Tilburg, The Netherlands; 2 Department of Epidemiology, GROW School for Oncology and Developmental Biology, Maastricht University, P. Debyeplein 1, 6200 MD Maastricht, The Netherlands; 3 Department of Surgery, Elkerliek Hospital, Wesselmanlaan 25, 5707 HA Helmond, The Netherlands; 4 Department of Internal Medicine and Gastroenterology, Zuyderland Medical Centre, Dr. H. van der Hoffplein 1, 6162 BG Sittard-Geleen, The Netherlands; 5 Department of Surgery, Maastricht University Medical Centre, P. Debyelaan 25, 6229 HX Maastricht, The Netherlands; 6 Nutrim School of Nutrition and Translational Research in Metabolism, Maastricht University, Maastricht, The Netherlands; 7 Departments of Surgery and Oncology, Máxima Medical Center, Postbus 7777, 5500 MB Veldhoven, The Netherlands; 8 Department of Surgery, VieCuri Medical Center, Postbus 1926, 5900 BX Venlo, The Netherlands; 9 Department of Research, Netherlands Comprehensive Cancer Organization (IKNL), Utrecht, The Netherlands

**Keywords:** Colorectal cancer, Cancer survivors, Alcohol drinking, Longitudinal studies

## Abstract

**Purpose:**

Alcohol consumption can lead to worse prognosis and mortality among colorectal cancer (CRC) patients. We investigated alcohol consumption of CRC survivors up to 2 years post-diagnosis, and how sociodemographic, lifestyle, and clinical factors were associated longitudinally with these habits.

**Methods:**

We pooled longitudinal data of 910 CRC survivors from the ongoing PROCORE and EnCoRe studies with data collected at diagnosis (baseline) and 3, 6, 12, and 24 months post-diagnosis. Both studies assessed alcohol consumption, including beer, wine, and liquor. Generalized estimated equation models were used to examine changes over time in alcohol consumption and multivariable longitudinal associations of sociodemographic, lifestyle, and clinical factors with alcohol consumption.

**Results:**

At baseline, participants were on average 67 years old, 332 (37%) were female, and alcohol was consumed by 79%. Most survivors (68–71%) drank less at all follow-ups. Beer, wine, and liquor were consumed by 51%, 58%, and 25% at baseline, respectively, and these declined over time. Males consumed more alcohol, and higher education, more physical activity, and not having a (permanent) stoma were associated with consuming more alcohol.

**Conclusion:**

CRC survivors decreased their alcohol consumption in the 2 years post-diagnosis. Future studies should take the significant factors that were associated with alcohol post-diagnosis consumption into account, when they investigate CRC health outcomes or for identifying subgroups for interventions. Males with higher education, more physical activity, and no stoma should be reminded after diagnosis for reducing their alcohol consumption.

**Supplementary Information:**

The online version contains supplementary material available at 10.1007/s00520-021-06104-0.

## Introduction

Colorectal cancer (CRC) is the second most common occurring cancer with ~ 500,000 cases, the cause of 43,000 cancer deaths in Europe each year [1], and the world’s fourth most deadly cancer type [2]. As a result of rising survival rates due to improved treatments and implementation of screening programs, the number of CRC survivors is increasing [3]. Alcohol consumption is a major risk factor for CRC [4] due to ethanol-induced DNA damage, epigenetics, and diet as potential causal mechanisms [5]. It has been suggested that alcohol may have a subsite-specific effect, affecting the rectum more than the colon [6]. A systematic analysis within the Global Burden of Disease Study found that the risk of all-cause mortality, and specifically of cancers, rises with increasing levels of alcohol consumption, and that for global disease burden and overall health, it is best not to consume at all [7]. Also, according to the World Cancer Research Fund/American Institute for Cancer Research (WCRF/AICR) report on lifestyle and cancer in 2018, all alcohol is detrimental to cancer and its prognosis [8]. The report makes no differences in cancer risk between the alcoholic beverage types (i.e., beer, wine, and liquor) [8]. Furthermore, this report recommends that cancer survivors (including CRC) should not consume any alcohol or, if they do, to limit it to national guidelines [8]. However, this recommendation is predominantly based on studies focusing on the risk of getting a cancer diagnosis, and includes only limited evidence on survivorship or mortality. Currently, little evidence is available regarding alcohol consumption and its determinants after a CRC diagnosis and how this is related to prognostic outcomes [9].

Some studies on alcohol consumption have been performed in cancer survivors, yet only a few have been longitudinal. To our knowledge, only a small number of studies looked at post-diagnosis alcohol consumption in CRC survivors, and these studies did not find an association with mortality [10–12]. Overall, these studies looked at alcohol only measured at one time point, instead of assessing changes over time in alcohol consumption from diagnosis until years later. Van Zutphen et al. looked at lifestyle changes in CRC survivors, and whether they were in concordance with the WCRF/AICR lifestyle recommendations for cancer prevention during that period: they reported a slight drop in total alcohol use 6 months post-diagnosis, then an increase again almost until baseline values at 2 years post-diagnosis [13]. In order to know more about the health effects of alcohol consumption in CRC survivors and the effects of alcohol consumption on further pathogenesis of CRC, it is important to first examine how consumption of alcohol and beverages is changing over time and which characteristics of CRC survivors are associated with such changes.

It is important to consider the influences of sociodemographic, lifestyle, and clinical characteristics on consumption of alcohol and specific beverage types. Firstly, insight into determinants of alcohol consumption after CRC may be used in future studies to facilitate the identification of subgroups at which lifestyle advice regarding alcohol consumption could be targeted after diagnosis and treatment. Secondly, these insights can be taken into account by clinicians who advocate less alcohol consumption to survivors. For instance, Park et al. have looked at the associations between alcohol use with sociodemographic, lifestyle, and some disease factors, but have not looked at specific beverage types, and their analyses were cross-sectional [14]. Also, consumption was significantly associated with mortality in 603 Chinese rectal cancer patients after diagnosis, compared to non-drinkers, but not in colon cancer patients [15]. It also needs to be elucidated in CRC survivors how alcohol consumption over time is influenced by treatment factors, such as receiving chemotherapy or radiotherapy, and having a stoma. Overall, the majority of the research performed up until now was cross-sectional and has hardly focused on separate beverage types.

Therefore, we aimed to study longitudinal changes in alcohol consumption in CRC survivors from diagnosis until 24 months post-diagnosis, both total alcohol consumption and consumption of beer, wine, and liquor as separate alcoholic beverages. We also aimed to analyze how sociodemographic, lifestyle, and clinical factors are longitudinally associated with alcohol consumption. Future studies should take these characteristics into account when they investigate the effect of alcohol on health outcomes or for identifying subgroups at which lifestyle advice regarding alcohol consumption could be targeted after diagnosis and treatment.

## Materials and methods

### Setting and participants

**Fig. 1:**
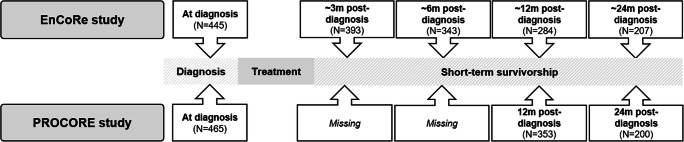
Schematic representation of the two datasets and how they were pooled

We used data from EnCoRe and PROCORE: two ongoing multi-center prospective cohort studies, in which CRC patients were included immediately after diagnosis, and followed up at several time points after treatment. We pooled the datasets and harmonized the time points as shown in Fig. 1.

#### “Energy for life after ColoRectal cancer” study

Energy for life after ColoRectal cancer (EnCoRe) is an ongoing multi-center prospective cohort study for which adult stage I–III CRC patients were enrolled at diagnosis and followed up at 6 weeks, 6 months, and 1 and 2 years after treatment, which is described in more detail elsewhere [16]. Patients were recruited at three hospitals in the Southeast of the Netherlands. Patients were not eligible in case of stage IV disease and comorbidities obstructing successful participation (e.g., cognitive disorders such as Alzheimer disease). The study has been registered at the Netherlands Trial Registry (www.trialregister.nl, NL6904) and approved by the Medical Ethics Committee of the University Hospital Maastricht and Maastricht University (approval number NL38786.068.11) in the Netherlands. Informed consent was obtained from all participants. Exclusion criteria were ever being diagnosed with stage IV CRC, having comorbidities that would obstruct successful participations, being unable to read or write Dutch, and having a home address outside the Netherlands. For the present analyses, we used data collected from April 2012 until July 2018. The post-treatment follow-ups correspond to approximately 3, 6, 12, and 24 months post-diagnosis. In total, we used data of 445 participants who provided data on the alcohol variables at baseline (Fig. 1).

#### “Patient-reported outcomes ColoRectal cancer” study

The patient-reported outcomes ColoRectal cancer (PROCORE) study, which started in 2016, is a prospective population-based study, in which 478 newly diagnosed stage I–IV adult CRC patients were recruited after diagnosis and the follow-ups were after surgery and until 12 and 24 months post-diagnosis via the PROFILES registry (Patient-Reported Outcomes Following Initial treatment and Long term Evaluation of Survivorship) [17]. Ethical approval for the study was obtained from the certified Medical Ethics Committee of Medical Research Ethics Committees United (approval number NL51119.060.14). All respondents gave informed consent. Patients were recruited from four Dutch hospitals in the south of the Netherlands. Exclusion criteria were ever being diagnosed with a different carcinoma, except for basal cell carcinoma of the skin, and having cognitive limitations or being unable to read or write Dutch, which did not allow them to independently fill out a questionnaire. For the current manuscript, we did not use the follow-up after surgery, as we did not measure alcohol consumption at that time point. The follow-ups were at 12 and 24 months post-diagnosis. Of all other time points, we used data of 465 participants that entered their alcohol consumption at baseline.

### Alcohol consumption

In EnCoRe, pre-diagnosis alcohol consumption was assessed retrospectively at diagnosis with a Food Frequency Questionnaire (FFQ). During follow-up, 7-day food diaries were used to assess alcohol consumption and intake of specific alcoholic beverages over the past week [18]. The validity of the FFQ over the past year has been evaluated relative to 7-day food diaries, and the intake of alcohol was highly correlated between both methods (rho = 0.91) [19]. In PROCORE, alcohol consumption was recorded with a questionnaire about the average frequency of alcohol consumption per day in the past year, and the number of glasses of beer, wine, and liquor.

Alcohol consumption was defined as follows: (A) alcohol consumption (y/n); (B) the number of drinks per week; (C) consuming beer, wine, or liquor; (D) the number of beer, wine, or liquor drinks per week; and (E) the categories of non-drinkers, moderate drinkers (< 14 drinks per week), and heavy drinkers (≥ 14 drinks/week), as provided in the Dutch guidelines for good nutrition of the Health Council of the Netherlands [20]. For each alcoholic drink, we assumed that all types of alcoholic beverages, i.e., beer (5% alcohol in 250 mL), wine (12% alcohol in 100 mL), or liquor (35% alcohol in 35 mL), contain 10 g ethanol per unit of consumption [20].

### Sociodemographic and lifestyle factors

In both cohorts, information was collected about age, sex, partner (yes/no), and working status (yes/no). Education levels were recorded as having low education (lower vocational and primary education), medium (intermediate vocational and secondary education), and high level (higher vocational and university). Participants’ level of moderate-to-vigorous physical activity (MVPA, hours/week) was measured by the Short QUestionnaire to ASsess Health-enhancing physical activity (SQUASH) [21]. Information was available on self-reported smoking (non-smoker, former, and current smoker), and body height and weight to determine body mass index (BMI, in kg/m^2^). Body height and weight were measured by trained dieticians in the EnCoRe study, while they were self-reported in the PROCORE study.

### Clinical factors

Both studies collected information from medical records on tumor localization (colon vs. rectum) and stage (I vs. II vs. III or IV), and treatments received besides surgery (chemotherapy and radiotherapy). Furthermore, the placement of a stoma was self-reported. The Self-Administered Comorbidity Questionnaire (SCQ) was used to assess the number of comorbidities (0, 1, ≥ 2) [22].

### Statistical analyses

All variables were described as percentages or means and standard deviations, or medians and interquartile ranges for non-normally distributed factors.

To analyze how alcohol consumption changed over time, we looked at the following: (A) alcohol consumption (y/n); (B) the number of drinks per week; (C) consuming beer, wine, or liquor; (D) the number of beer, wine, or liquor drinks per week; and (D) the categories of non-drinkers, moderate, and heavy drinkers for total alcohol, beer, wine, and liquor. We compared the categories at each time point, and assessed how survivors changed in alcohol consumption category from baseline towards 12 months post-diagnosis.

In order to determine longitudinal associations of sociodemographic, lifestyle, and clinical factors with alcohol consumption over time, we used generalized estimating equations (GEE) with an exchangeable correlation structure, which takes into account within-person correlations when examining multiple observations per subject and can handle missing values [23]. Alcohol consumption is considered as a non-transformed outcome (link = Identity), and all sociodemographic, lifestyle, and clinical factors as determinants, while also correcting for cohort. We performed additional analyses in which we ran the same analyses with the number of alcoholic drinks per week as the outcome including only baseline and the time points 12 and 24 months post-diagnosis, for which we had data from both cohorts. All analyses were conducted using SPSS version 24.0 (IBM Corp., Armonk, NY, USA). Significance level was set at *p* < 0.05, two-tailed.

## Results

### Sample characteristics

Study participants (*n* = 910) were on average 67 years old at diagnosis, and 37% were female (Table 1). CRC patients in PROCORE showed a slightly lower education level, more often had a partner, more often adhered to physical activity guidelines, had lower BMI, were more often diagnosed with colon instead of rectum cancer, and were less often treated with chemo- or radiotherapy compared to patients in EnCoRe. Supplementary Tables 1A and 1B show the characteristics of both the PROCORE and EnCoRe samples at each time point.

**Table 1:** Baseline characteristics of total sample, and the EnCoRe vs. PROCORE datasets

	Total (*N* = 910)	EnCoRe (*N* = 445)	PROCORE (*N* = 465)
Sociodemographics (*N* (%))			
Age (years, mean (SD))	66.9 (9.2)	66.7 (9.1)	67.0 (9.2)
Sex (females)	332 (36.5)	151 (33.9)	181 (38.9)
Education level			
High	270 (29.7)	149 (33.5)	121 (26.4)
Medium	556 (61.5)	266 (59.8)	290 (63.2)
Low	78 (8.6)	30 (6.7)	48 (10.5)
Having a partner	752 (82.7)	356 (80.0)	396 (85.3)
Working	290 (32.6)	148 (33.3)	142 (31.9)
Lifestyle factors (median (IQR))			
Physical activity (hours/week MVPA) ^a^	11 (12)	11 (14)	11 (11)
Smoking categories			
Never	273 (30.0)	139 (31.2)	134 (28.9)
Former	527 (58.0)	252 (56.6)	275 (59.3)
Current	109 (12.0)	54 (12.1)	55 (11.9)
BMI (kg/m^2^, mean (SD))	27.4 (4.4)	28.3 (4.7)	26.6 (4.0)
Clinical factors (*N* (%)			
Months since diagnosis (median (range))	0.6 (0–11)	0.5 (0–5)	0.7 (0–11)
Tumor location			
Colon	604 (66.4)	281 (63.1)	323 (69.5)
Rectum	303 (33.3)	164 (36.9)	139 (29.9)
Staging			
I	254 (29.0)	122 (29.0)	132 (29.0)
II	236 (27.0)	102 (24.3)	134 (29.5)
III	367 (41.9)	196 (46.7)	171 (37.6)
IV	18 (2.1)	-	18 (4.0)
Chemotherapy	325 (35.7)	178 (40.0)	147 (31.6)
Radiotherapy	194 (21.3)	112 (25.2)	82 (17.6)
Stoma placement	3 (0.3)	0 (0)	3 (0.7)
Number of comorbidities ^b^			
None	120 (26.2)	-	120 (26.2)
1	157 (34.3)	-	157 (34.3)
≥2	181 (39.5)	-	181 (39.5)

^a^*MVPA*, moderate-to-vigorous physical activity

^b^Comorbidities are only measured from 3 months post-diagnosis follow-up in EnCoRe

### Changes over time in alcohol consumption

At baseline, 79% of the participants reported consuming alcohol (Table 2). At 3 and 6 months post-diagnosis, 68% was still consuming, and the percentages of drinkers were 71% and 70% after 12 and 24 months, respectively. On average, survivors drank 8.4 drinks per week at diagnosis, which decreased to 7.8 drinks per week at 24 months post-diagnosis. At baseline, 56% consumed moderate amounts and 23% were heavy drinkers (Supplementary Table 2). Throughout the follow-up, there seemed to be less moderate drinkers, and more non-drinkers. Supplementary Table 3 shows the changes in categories from baseline towards 12 months post-diagnosis.

**Table 2:** Alcohol consumption at each follow-up in the pooled EnCoRe and PROCORE cohorts, and changes relative to consumption at diagnosis (baseline)

	EnCoRe + PROCORE	EnCoRe		EnCoRe		EnCoRe + PROCORE		EnCoRe + PROCORE	
Alcohol consumption (means (SD) and *N* (%))	At diagnosis (*N* = 910)	3 m post-diagnosis (*N* = 381)	*p*-value vs. baseline ^b^	6 m post-diagnosis (*N* = 329)	*p*-value vs. baseline ^b^	12 m post-diagnosis (*N* = 620)	*p*-value vs. baseline ^b^	24 m post-diagnosis (*N* = 392)	*p*-value vs. baseline ^b^
Drinking alcohol (y/n)	719 (79.0)	259 (68.0)	**< .001**	225 (68.4)	**< .001**	441 (71.1)	**< .001**	276 (70.4)	**< .001**
Total (drinks/week) ^a^	8.4 (0.4)	8.1 (0.5)	.48	8.4 (0.5)	.95	8.0 (0.4)	.09	7.8 (0.4)	**.03**
Drinking beer (y/n)	460 (50.5)	162 (42.5)	**< .001**	143 (43.5)	**< .001**	242 (39.0)	**< .001**	160 (40.8)	**< .001**
Beer (drinks/week) ^a^	4.0 (0.3)	3.9 (0.4)	.87	4.0 (0.4)	.96	3.9 (0.3)	.64	3.8 (0.3)	.51
Drinking wine (y/n)	526 (57.8)	177 (46.5)	**< .001**	155 (47.1)	**< .001**	306 (49.4)	**< .001**	195 (49.7)	**< .001**
Wine (drinks/week) ^a^	3.7 (0.2)	3.4 (0.2)	.10	3.5 (0.2)	.31	3.4 (0.2)	**.03**	3.4 (0.2)	.07
Drinking liquor (y/n)	231 (25.4)	94 (24.7)	**< .001**	71 (21.6)	**< .001**	115 (18.5)	**< .001**	77 (19.6)	**< .001**
Liquor (drinks/week) ^a^	0.8 (0.1)	0.8 (0.1)	.59	0.9 (0.1)	.16	0.7 (0.1)	.64	0.6 (0.1)	**.04**

*P*-values < 0.05 are represented bold

*SD*, standard deviations

^a^One drink consists of 10 g alcohol

^b^Changes in drinks were calculated with GEE analyses, corrected for time point and cohort, and *p*-values represent changes compared to baseline

When we investigated beer, wine, and liquors, there were 51%, 58%, and 25% drinkers at baseline, respectively. During follow-up, less survivors were consuming beer, wine, and liquor, as compared to baseline, and there were 41%, 50%, and 20% drinkers at the last follow-up (Table 2). The amount of drinks of each beverage remained relatively constant over time, with survivors reporting to drink roughly 4 beers per week, 3–4 wines per week, and 1 glass of liquor per week. Furthermore, the majority were non-drinkers or moderate drinkers at all time points, and the number of heavy liquor drinkers was very low (Supplementary Table 2).

### Associations of sociodemographic, lifestyle, and clinical factors with alcohol consumption

Table 3 shows longitudinal associations between sociodemographic, lifestyle, and clinical factors with alcohol use in multivariable analyses. Women reported consuming less alcohol per week than men over time (Supplementary Fig. 1). Additionally, a lower education level and the presence of a stoma were associated with less alcohol consumption, whereas more physical activity was related to more alcohol consumption over time.

**Table 3:** Longitudinal multivariable associations of sociodemographic, lifestyle, and clinical factors with total alcoholic drinks and types of drinks per week

	Alcohol drinks/week		Beer drinks/week		Wine drinks/week		Liquor drinks/week	
	B (SE)	*p*	B (SE)	*p*	B (SE)	*p*	B (SE)	*p*
Cohort (ref = PROCORE)	1.13 (0.81)	.16	1.62 (0.67)	**.02**	0.13 (0.40)	.74	− 0.24 (0.19)	.20
Sociodemographics								
Baseline age	− 0.04 (0.05)	.36	− 0.11 (0.04)	**.003**	0.03 (0.02)	.14	0.03 (0.01)	**.02**
Sex (ref = male)	− 6.18 (0.72)	**< .001**	− 5.64 (0.50)	**<.001**	0.33 (0.39)	.40	− 0.46 (0.18)	**.01**
Education levels (ref = high)								
Medium	− 2.85 (1.17)	**.003**	0.99 (0.70)	.16	− 3.36 (0.49)	**< .001**	− 0.50 (0.28)	.07
Low	− 5.36 (0.94)	**< .001**	0.53 (0.88)	.55	− 4.88 (0.58)	**< .001**	− 0.84 (0.39)	**.03**
Having a partner (ref = no)	− 1.32 (0.91)	.15	− 0.31 (0.61)	.61	− 0.15 (0.35)	.67	0.03 (0.32)	.93
Working (ref = no work)	0.90 (0.92)	.33	0.38 (0.91)	.68	0.25 (0.38)	.51	− 0.24 (0.19)	.21
Lifestyle								
Physical activity (hours)	0.05 (0.02)	**.03**	0.02 (0.02)	.26	0.03 (0.01)	**.01**	0.02 (0.01)	**.02**
Body mass index (kg/m^2^)	0.30 (0.18)	.08	0.06 (0.14)	.64	0.03 (0.06)	.65	0.07 (0.02)	**.002**
Smoking categories (ref = never)								
Former smoker	− 0.05 (0.77)	.95	− 0.03 (0.34)	.94	0.95 (0.43)	**.03**	− 0.01 (0.18)	.95
Current smoker	− 2.23 (2.24)	.32	− 1.11 (0.86)	.20	0.98 (0.72)	.17	0.29 (0.39)	.46
Clinical factors								
Months since diagnosis ^a^	− 0.04 (0.02)	**.03**	− 0.01 (0.01)	.56	− 0.02 (0.01)	**.04**	− 0.01 (0.00)	**.002**
Tumor location (ref = colon cancer)	1.22 (1.13)	.28	0.59 (0.97)	.55	0.46 (0.61)	.45	0.09 (0.26)	.74
Tumor staging (ref = stage I)								
Stage II	0.19 (1.04)	.86	0.20 (0.88)	.82	− 0.11 (0.49)	.82	0.07 (0.26)	.78
Stage III or IV	− 0.96 (1.09)	.38	− 1.35 (0.80)	.09	1.17 (0.60)	**.05**	− 0.47 (0.36)	.19
Chemotherapy (ref = no)	0.76 (1.11)	.49	0.85 (0.81)	.30	− 1.18 (0.56)	**.04**	0.80 (0.38)	**.03**
Radiotherapy (ref = no)	− 0.02 (1.40)	.99	− 0.40 (1.07)	.71	− 0.05 (0.76)	.95	0.07 (0.40)	.87
Stoma placement (ref = no)	− 2.21 (0.75)	**.003**	− 1.33 (0.66)	**.05**	− 0.84 (0.31)	**.01**	0.09 (0.21)	.67
Number of comorbidities (ref = none)								
1	− 0.26 (0.59)	.67	0.06 (0.43)	.90	− 0.61 (0.35)	.08	0.07 (0.17)	.70
≥ 2	− 0.04 (0.73)	.96	− 0.11 (0.53)	.84	− 0.62 (0.38)	.11	0.06 (0.19)	.76

*P*-values < 0.05 are represented bold

^a^Months since diagnosis was entered into the models as a measure of time

Survivors in the EnCoRe study reported more beer consumption than survivors in the PROCORE study. Survivors with lower age at baseline were consuming more beer, men drank almost six glasses per week more than women, and survivors with a stoma drank less beer. More wine consumption was associated with higher education, more physical activity, and former smoking. Survivors with tumor stage III or IV drank more wine, whereas chemotherapy and presence of a stoma were associated with less wine consumption. For use of liquor, higher baseline age, being male, having a higher education, being more physically active, having a higher BMI, and having received chemotherapeutic treatment were associated with higher consumption.

### Additional analyses

We ran the analyses using only baseline, and the 12 and 24 months post-diagnosis time points. Survivors in the EnCoRe study consumed more alcohol than survivors in the PROCORE study. Although most associations were consistent with the main analyses, now we saw that both former and current smoking were associated with consuming two or three glasses more per week, respectively, and a stoma placement was not associated with alcohol consumption anymore (Supplementary Table 4).

## Discussion

In this pooled sample of two Dutch CRC survivor cohorts, we observed that alcohol consumption is slightly decreasing after diagnosis, and remains lower up to 2 years post-diagnosis. Over time, we found that survivors who were male, with higher education, more physical activity, and survivors without a stoma reported higher consumption of alcoholic drinks.

At baseline, we found that our study population had more alcohol consumers than reported in other cancer survivor studies: 49% current consumers [14], 8% moderate consumers [24], and 88% low alcohol consumption (≤ 1 drink/day) [25], but all alcoholic consumptions seemed to decrease slightly in the 2 years post-diagnosis. This was in line with the paper of Bours et al. stating that 95% drank less alcohol after CRC diagnosis in a cross-sectional study 7 years post-diagnosis [26]. They saw that alcohol use did not change from the first (roughly 5 years after diagnosis) to the third survey of an ongoing longitudinal study in CRC survivors, and concluded that these changes had probably occurred closer to diagnosis [26]. Even though we see this decrease in the general older population of the Netherlands [27], their reasons for consuming less alcohol may be different than for CRC survivors in whom the reasons may be related to prevention of cancer recurrence, to improve health, and prevent disease in general, as Bours et al. state [26].

Of the sociodemographic and lifestyle factors, we found that male sex, a higher education level, and being more physically active were related to higher alcohol consumption. We found that men were consuming more alcohol than women at every time point, in line with other studies [28]. Cortés-Ibáñez et al. have found in 5473 Dutch cancer survivors (± 9 years post-diagnosis) that after adjustments for age and sex, cancer survivors drank less alcohol, more often had normal weight, and were more physically active than the non-cancer participants, pointing to an improved lifestyle after cancer diagnosis. Another large cohort including 1153 Korean cancer survivors (59% diagnosed within the past 5 years, and 19% > 10 years) also found that (heavy) drinkers were younger, men, and had a higher household income [14]. However, they did find that drinkers were more often current smokers, and there was no association with education or physical activity [14]. Possibly, higher household income was a better marker for socioeconomic status than education in the Korean cohort. Also, alcoholic drinks were not related to smoking in our pooled cohort, only former smoking was related to wine drinking. Moreover, when we ran the analyses without the first follow-ups (3 and 6 months post-diagnosis), we did see that both former and current smokers drank more alcohol. Lastly, the Korean study included walking regularly as being physically active, and our definition of physical activity adherence seems to be more rigorous, and is associated with consuming more. In general, it seems that cancer survivors tried to lead a better lifestyle, potentially driven by their diagnosis and treatment as “teachable moments” that motivated them to make behavioral changes for improving their health [29]. Breedveld-Peters et al. also reported that 64% of 155 CRC survivors were adhering to alcohol guidelines of the WCRF/AICR 6 years post-diagnosis, but still 18% had low adherence [30]. Perhaps a better result may be achieved if more people would be aware of the alcohol guidelines, as a study from California showed that 15% of CRC survivors had never heard of recommendations to limit alcohol [31].

Overall, alcohol consumption may be influenced by local culture. In the Netherlands, persons above 55 years reported to drink in order to relax, to sleep better, or to have less pain or because they believe it is good for health [27]. In interviews among the elderly, 75% of persons above 55 years did not know that alcohol is addictive, and 83% did not know about its disadvantages for health [27]. Persons reported that feeling lonely, having lost structure in life (e.g., due to retirement) or have experienced large stress (e.g., CRC diagnosis in our sample), special occasions or social events may be reasons for consuming alcohol [27].

In the specific analyses among beverage subtypes, we observed an association between chemotherapy and consuming less wine and more liquors. Survivors with stage III or IV CRC reported more wine consumption over time. Also, we found an association between patients with a stoma and less consumption of alcohol, specifically less beer or wine. This may be due to some of the problems these survivors encounter, such as a lower quality of life, more concerns about their illness, and a higher health care consumption compared with those without a stoma 1–10 years after diagnosis [32], sexual problems, or depressive feelings [33]. According to the guidelines in the Netherlands for patients with a colostomy, they should be careful with drinking carbonated drinks (e.g., beer) due to its effect on flatulence [34]. Furthermore, some papers found that heavy drinking was associated with a risk of anastomotic leaks [35] or parastomal bulging [36].

The results of our study should be interpreted while taking into account the following limitations. Firstly, it is important to recognize that each study uses different cut-offs for moderate and heavy drinking. Some studies defined moderate alcohol consumption as 5–15 g per day for females and 5–30 g per day for males [24], and others state that heavy consumption is ≥ 60 g alcohol (seven drinks) for males and ≥40 g (five drinks) for females, twice or more per week [14]. We currently use the guidelines for good nutrition of the Health Council of the Netherlands, stating that 14 drinks per week are defined as moderate alcohol consumption [20]. Furthermore, alcohol consumption was measured both with the FFQ over the past year and 7-day food diaries, which may limit the harmonization of the two datasets. Nevertheless, the intake of alcohol was highly correlated between both methods (rho = 0.91) [19]. Next, we pooled and harmonized two studies that had slightly different time points at follow-up after diagnosis. Therefore, all analyses were corrected for months since diagnosis. In addition, we performed additional analyses, merely analyzing the similar time points (baseline, 12 months, and 24 months), and these results were largely consistent. Another limitation was that all lifestyle factors were self-reported, and hence, we cannot exclude the possibility that participants answered questions in a socially desirable manner.

An important strength of the present study is that pooled analyses were performed using two longitudinal databases with repeated measurements of survivors prospectively followed up for a period of approximately 2 years from the moment of CRC diagnosis. Most other studies included patients longer after diagnosis or performed cross-sectional analyses.

## Conclusions

To our knowledge, this is the first study in CRC survivors that longitudinally analyzed consumption of alcohol and specific alcoholic beverages in relation to sociodemographic, lifestyle, and clinical factors. From CRC diagnosis until 2 years post-diagnosis, we found that most survivors are modestly decreasing their alcohol consumption. Several sociodemographic, lifestyle, and clinical factors were related to alcohol consumption over time, including sex, education level, physical activity, and having a stoma. The findings give insight into characteristics of CRC survivors that use alcohol, which should be taken into account in future analyses of alcohol on cancer outcomes.

## Supplementary Information

Supplementary Fig. 1 Mean alcoholic drinks per week for all survivors and for men vs. women (PNG 209 kb)

High resolution image (TIF 78 kb)

ESM 1 (DOCX 48 kb)

## Data Availability

The data that support the findings of this study are available on request from the corresponding author. The data are not publicly available due to privacy or ethical restrictions.
