# Supplementary figures and images for: Longitudinal associations of sociodemographic, lifestyle, and clinical factors with alcohol consumption in colorectal cancer survivors up to 2 years post-diagnosis
Source: Support Care Cancer. 2021 Mar 24;29(10):5935–43. doi: 10.1007/s00520-021-06104-0 (PMC8410706; doi:10.1007/s00520-021-06104-0)

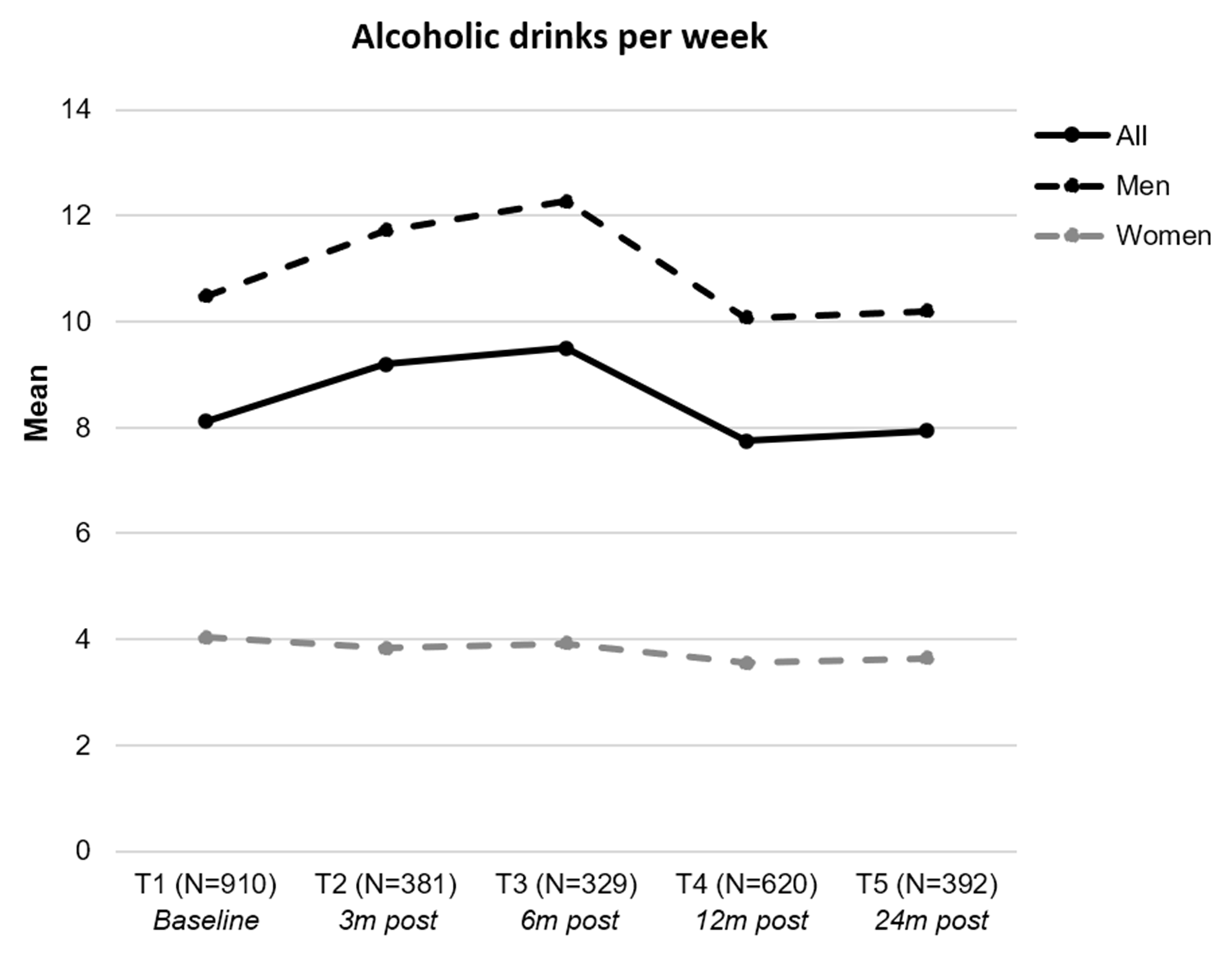

Supplement: Supplementary file 1 — Mean alcoholic drinks per week for all survivors and for men vs. women (PNG 209 kb) [file 520_2021_6104_Fig2_ESM.png]

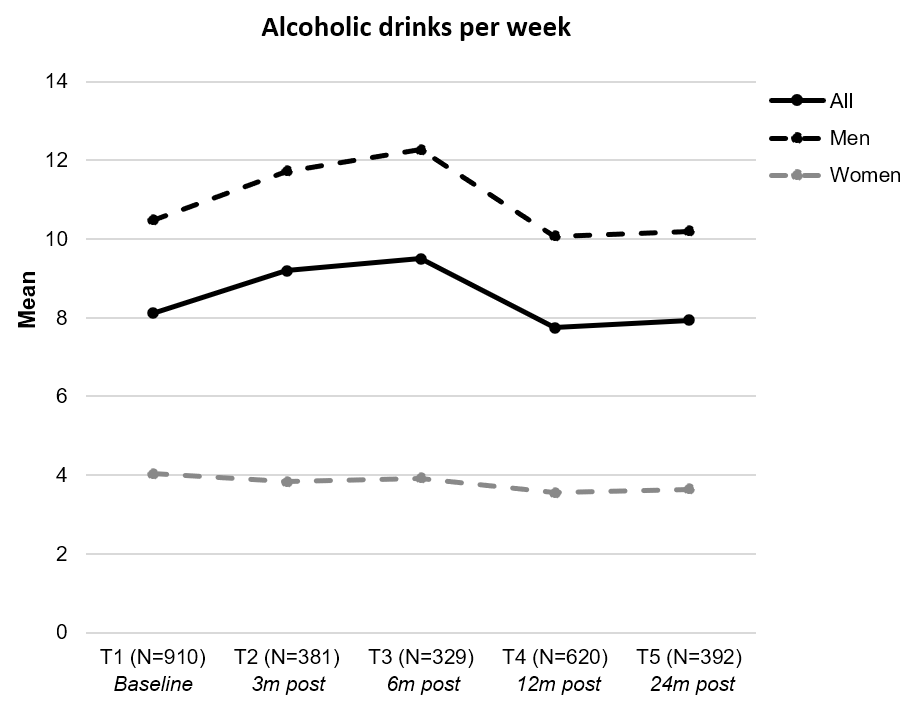

Supplement: Supplementary file 2 — High resolution image (TIF 78 kb) [file 520_2021_6104_MOESM1_ESM.tif]
