# Supplementary material for: Longitudinal associations of sociodemographic, lifestyle, and clinical factors with alcohol consumption in colorectal cancer survivors up to 2 years post-diagnosis
Source: Support Care Cancer. 2021 Mar 24;29(10):5935–43. doi: 10.1007/s00520-021-06104-0 (PMC8410706; doi:10.1007/s00520-021-06104-0)
Supplement: Supplementary file 3 — (DOCX 48 kb) [file 520_2021_6104_MOESM2_ESM.docx]

**Supplementary Tables for:**

**Longitudinal associations of sociodemographic, lifestyle and clinical factors with alcohol consumption in colorectal cancer survivors up to 2 years post-diagnosis**

Dóra Révész^1^, Martijn J.L. Bours^3^, J.A. Wegdam^4^, Eric T.P. Keulen^5^, Stéphanie O. Breukink^3,6,7^, Gerrit D. Slooter^8^, Jeroen Vogelaar^9^, Matty P. Weijenberg^3^, Floortje Mols^1,2^.

^1^ Center of Research on Psychological and Somatic disorders (CoRPS), Department of Medical and Clinical Psychology, Tilburg University, Tilburg, The Netherlands

^2^ Department of Research, Netherlands Comprehensive Cancer Organization (IKNL), Utrecht, The Netherlands

^3^ Department of Epidemiology, GROW School for Oncology and Developmental Biology, Maastricht University, Maastricht, the Netherlands

^4^ Department of Surgery, Elkerliek Hospital, Helmond, The Netherlands

^5^ Department of Internal Medicine and Gastroenterology, Zuyderland Medical Centre, Sittard-Geleen, The Netherlands

^6^ Department of Surgery, Maastricht University Medical Centre, Maastricht, the Netherlands

^7^ Nutrim School of Nutrition and Translational Research in Metabolism, Maastricht University, Maastricht, the Netherlands

^8^ Departments of Surgery and Oncology, Máxima Medical Center, Veldhoven, The Netherlands

^9^ Departments of Surgery, VieCuri Medical Center, Venlo, The Netherlands

**Corresponding author**

Dóra Révész, PhD

CoRPS, Department of Medical and Clinical Psychology, Tilburg University, PO Box 90153, 5000 LE Tilburg, the Netherlands, D.Revesz@tilburguniversity.edu

| **Supplementary Table 1A: Characteristics of EnCoRe sample at each time point** | | | | | | |
| --- | --- | --- | --- | --- | --- | --- |
|  | | After diagnosis (N=445) | 3m post-diagnosis (N=393) | 6m post-diagnosis (N=343) | 12m post-diagnosis (N=284) | 24m post-diagnosis (N=207) |
| Sociodemographics, N (%) | |  |  |  |  |  |
| Age (years, mean (SD)) | | 66.7 (9.1) | 66.9 (9.1) | 67.2 (9.2) | 67.3 (9.2) | 68.0 (9.1) |
| Sex (females) | | 151 (33.9) | 126 (32.1) | 111 (32.4) | 92 (32.4) | 65 (31.4) |
| Education level | |  |  |  |  |  |
|  | High | 149 (33.5) | 137 (34.9) | 117 (34.1) | 98 (34.5) | 73 (35.3) |
|  | Medium | 266 (59.8) | 231 (58.8) | 205 (59.8) | 170 (59.9) | 126 (60.9) |
|  | Low | 30 (6.7) | 25 (6.4) | 21 (6.1) | 16 (5.6) | 8 (3.9) |
| Having a partner | | 356 (80.0) | 305 (79.4) | 269 (80.3) | 222 (80.1) | 160 (81.2) |
| Working | | 148 (33.3) | 113 (29.4) | 92 (27.1) | 78 (27.8) | 47 (23.6) |
| Lifestyle (median (IQR)) | |  |  |  |  |  |
| Physical activity (hrs/wk MVPA) ^a^ | | 11 (13.5) | 7 (10.6) | 9.5 (11) | 10 (12.5) | 10 (12.5) |
| Smoking categories | |  |  |  |  |  |
|  | Never | 139 (31.2) | 118 (30.7) | 98 (28.9) | 78 (27.8) | 57 (28.8) |
|  | Former | 252 (56.6) | 233 (60.7) | 212 (62.5) | 176 (62.6) | 122 (61.6) |
|  | Current | 54 (12.1) | 33 (8.6) | 29 (8.6) | 27 (9.6) | 19 (9.6) |
| BMI (kg/m^2^, mean (SD)) | | 28.3 (4.7) | 27.7 (4.5) | 28.2 (4.7) | 28.6 (4.8) | 28.4 (4.6) |
| Clinical factors (N (%)) | |  |  |  |  |  |
| Months since diagnosis (median (range)) | | 0.5 (0-5) | 3.2 (1-13) | 7.5 (5-17) | 13.4 (11-23) | 25.5 (23-36) |
| Tumor location | |  |  |  |  |  |
|  | Colon | 281 (63.1) | 247 (62.8) | 219 (63.8) | 179 (63.0) | 124 (59.9) |
|  | Rectum | 164 (36.9) | 146 (37.2) | 124 (36.2) | 105 (37.0) | 83 (40.1) |
| Staging | |  |  |  |  |  |
|  | I | 122 (29.0) | 110 (29.6) | 97 (29.8) | 88 (32.2) | 66 (33.0) |
|  | II | 102 (24.3) | 96 (25.8) | 83 (25.5) | 66 (24.2) | 49 (24.5) |
|  | III | 196 (46.7) | 166 (44.6) | 146 (44.8) | 119 (43.6) | 85 (42.5) |
|  | IV | - | - | - | - | - |
| Chemotherapy | | 178 (40.0) | 155 (39.3) | 132 (38.5) | 107 (37.7) | 80 (38.6) |
| Radiotherapy | | 112 (25.2) | 101 (25.6) | 86 (25.1) | 73 (25.7) | 56 (27.1) |
| Stoma placement | | 0 (0) | 109 (28.4) | 66 (19.5) | 42 (14.9) | 26 (13.1) |
| Number of comorbidities ^b^ | |  |  |  |  |  |
|  | None | - | 90 (23.0) | 87 (25.4) | 70 (25.1) | 46 (22.8) |
|  | 1 | - | 101 (25.8) | 86 (25.1) | 63 (22.6) | 48 (23.8) |
|  | ≥2 | - | 201 (51.3) | 170 (49.6) | 146 (52.3) | 108 (53.5) |
| **Footnotes**:  a) MVPA = moderate-to-vigorous physical activity;  b) Comorbidities are only measured from 3 months post-diagnosis follow-up in EnCoRe. | | | | | | |

| **Supplementary Table 1B: Characteristics of PROCORE sample at each time point** | | | | | | |
| --- | --- | --- | --- | --- | --- | --- |
|  | | After diagnosis (N=465) | 3m post-diagnosis (N=0) | 6m post-diagnosis (N=0) | 12m post-diagnosis (N=353) | 24m post-diagnosis (N=200) |
| Sociodemographics, N (%) | |  |  |  |  |  |
| Age (years, mean (SD)) | | 67.0 (9.2) |  |  | 67.9 (8.8) | 67.7 (8.7) |
| Sex (females) | | 181 (38.9) |  |  | 135 (38.2) | 73 (36.5) |
| Education level | |  |  |  |  |  |
|  | High | 121 (26.4) |  |  | 98 (27.9) | 59 (29.6) |
|  | Medium | 290 (63.2) |  |  | 220 (62.7) | 122 (61.3) |
|  | Low | 48 (10.5) |  |  | 33 (9.4) | 18 (9.0) |
| Having a partner | | 396 (85.3) |  |  | 298 (85.1) | 173 (87.4) |
| Working | | 142 (31.9) |  |  | 95 (28.2) | 51 (27.3) |
| Lifestyle (median (IQR)) | |  |  |  |  |  |
| Physical activity (hrs/wk MVPA) ^a^ | | 11 (10.5) |  |  | 11.5 (13) | 11 (11) |
| Smoking categories | |  |  |  |  |  |
|  | Never | 134 (28.9) |  |  | 110 (31.3) | 62 (31.3) |
|  | Former | 275 (59.3) |  |  | 218 (62.1) | 122 (61.6) |
|  | Current | 55 (11.9) |  |  | 23 (6.6) | 14 (7.1) |
| BMI (kg/m^2^, mean (SD)) | | 26.6 (4.0) |  |  | 26.8 (4.0) | 27.1 (4.0) |
| Clinical factors (N (%)) | |  |  |  |  |  |
| Months since diagnosis (median (range)) | | 0.7 (0-11) |  |  | 12.9 (5-23) | 24.9 (18-29) |
| Tumor location | |  |  |  |  |  |
|  | Colon | 323 (69.5) |  |  | 248 (70.7) | 136 (68.3) |
|  | Rectum | 139 (29.9) |  |  | 103 (29.3) | 63 (31.7) |
| Staging | |  |  |  |  |  |
|  | I | 132 (29.0) |  |  | 111 (32.1) | 64 (32.3) |
|  | II | 134 (29.5) |  |  | 96 (27.7) | 52 (26.3) |
|  | III | 171 (37.6) |  |  | 129 (37.3) | 78 (39.4) |
|  | IV | 18 (4.0) |  |  | 10 (2.9) | 4 (2.0) |
| Chemotherapy | | 147 (31.6) |  |  | 108 (30.6) | 63 (31.5) |
| Radiotherapy | | 82 (17.6) |  |  | 57 (16.1) | 32 (16.0) |
| Stoma placement | | 3 (0.7) |  |  | 39 (11.2) | 16 (8.2) |
| Number of comorbidities | |  |  |  |  |  |
|  | None | 120 (26.2) |  |  | 109 (31.5) | 57 (28.9) |
|  | 1 | 157 (34.3) |  |  | 120 (34.7) | 57 (28.9) |
|  | ≥2 | 181 (39.5) |  |  | 117 (33.8) | 83 (42.1) |
| **Footnotes**: a) MVPA = moderate-to-vigorous physical activity. | | | | | | |

| **Supplementary Table 2: Distribution of categories of alcohol consumption at each follow-up in the pooled cohort** | | | | | | |
| --- | --- | --- | --- | --- | --- | --- |
|  | | After diagnosis (N=910) | 3m post-diagnosis (N=381) | 6m post-diagnosis (N=329) | 12m post-diagnosis (N=620) | 24m post-diagnosis (N=392) |
|  | | EnCoRe  +  PROCORE | EnCoRe | EnCoRe | EnCoRe  +  PROCORE | EnCoRe  +  PROCORE |
| Total alcohol (N (%)) ^a^ | |  |  |  |  |  |
|  | Non-drinker ^b^ | 191 (21.0) | 122 (31.9) | 104 (31.3) | 179 (28.9) | 118 (29.8) |
|  | Moderate ^b^ | 512 (56.3) | 174 (45.4) | 142 (42.8) | 318 (50.6) | 197 (49.7) |
|  | Heavy ^b^ | 207 (22.7) | 87 (22.7) | 86 (25.9) | 128 (20.3) | 81 (20.5) |
| Total beer (N (%)) ^a^ | |  |  |  |  |  |
|  | Non-drinker ^b^ | 450 (49.4) | 221 (57.7) | 187 (56.3) | 383 (60.9) | 234 (59.1) |
|  | Moderate ^b^ | 377 (41.4) | 125 (32.6) | 110 (33.1) | 190 (30.2) | 130 (32.8) |
|  | Heavy ^b^ | 83 (9.1) | 37 (9.7) | 35 (10.5) | 56 (8.9) | 32 (8.1) |
| Total wine (N (%)) ^a^ | |  |  |  |  |  |
|  | Non-drinker ^b^ | 384(42.2) | 204 (53.3) | 176 (53.0) | 319 (50.7) | 200 (50.5) |
|  | Moderate ^b^ | 455(50.0) | 147 (38.4) | 129 (38.9) | 263 (41.8) | 167 (42.2) |
|  | Heavy ^b^ | 71(7.8) | 32 (8.4) | 27 (8.1) | 47 (7.5) | 29 (7.3) |
| Total liquor (N (%)) ^a^ | |  |  |  |  |  |
|  | Non-drinker ^b^ | 679 (74.6) | 289 (75.5) | 259 (78.0) | 514 (81.7) | 319 (80.6) |
|  | Moderate ^b^ | 214 (23.5) | 91 (23.8) | 69 (20.8) | 107 (17.0) | 73 (18.4) |
|  | Heavy ^b^ | 17 (1.9) | 3 (0.8) | 4 (1.2) | 8 (1.3) | 4 (1.0) |
| **Footnotes**: a) One drink consists of 10gr alcohol; b) Persons were non-drinkers vs. moderate (<14 drinks/week) vs. heavy drinkers (≥14 drinks/week) | | | | | | |

| **Supplementary Table 3: Survivors changing in alcohol consumption categories from T1 to T4 in the pooled cohort** | | | | | |
| --- | --- | --- | --- | --- | --- |
| T1: After diagnosis (N=910) | | | T4: 12m post-diagnosis (N=620) | | |
|  | | N (%) | Non-drinker | Moderate | Heavy |
|  | Non-drinker ^a^ | 191 (21.0) | 113 (91.1) | 11 (8.9) | - |
|  | Moderate ^a^ | 512 (56.3) | 65 (18.5) | 260 (73.9) | 27 (7.7) |
|  | Heavy ^a^ | 207 (22.7) | 1 (0.7) | 42 (29.2) | 101 (70.1) |
| **Footnotes**: a) Persons were non-drinkers vs. moderate (<14 drinks/week) vs. heavy drinkers (≥14 drinks/week) | | | | | |

| **Supplementary Table 4: Longitudinal multivariable associations of sociodemographic, lifestyle and clinical factors with alcoholic drinks per week at T1, T4 and T5 only (time points available for both studies)** | | | | | | | | | |
| --- | --- | --- | --- | --- | --- | --- | --- | --- | --- |
|  | | **Alcohol drinks/week** | | **Beer drinks/week** | | **Wine drinks/week** | | **Liquor drinks/week** | |
|  | | **B (SE)** | **p** | **B (SE)** | **p** | **B (SE)** | **p** | **B (SE)** | **p** |
| Cohort (ref=PROCORE) | | 2.58 (0.97) | .**01** | 2.19 (0.79) | **.01** | 0.62 (0.47) | .19 | -0.29 (0.19) | .13 |
| **Sociodemographics** | |  |  |  |  |  |  |  |  |
| Baseline age | | -0.08 (0.05) | .12 | -0.14 (0.04) | **.002** | 0.03 (0.03) | .23 | 0.02 (0.01) | .11 |
| Sex (ref=male) | | -5.90 (0.75) | **<.001** | -5.60 (0.56) | **<.001** | 0.25 (0.42) | .55 | -0.46 (0.19) | **.02** |
| Education levels (ref=high) | |  |  |  |  |  |  |  |  |
|  | Medium | -2.48 (1.03) | **.02** | 0.95 (0.76) | .21 | -3.09 (0.56) | **<.001** | -0.40 (0.27) | .13 |
|  | Low | -4.39 (1.14) | **<.001** | 0.68 (0.89) | .44 | -4.52 (0.65) | **<.001** | -0.57 (0.39) | .15 |
| Having a partner (ref=no) | | -0.79 (0.99) | .42 | -0.86 (0.76) | .26 | -0.24 (0.51) | .64 | 0.28 (0.26) | .27 |
| Working (ref=no work) | | 0.36 (1.07) | .97 | 0.21 (0.88) | .81 | 0.05 (0.57) | .93 | -0.38 (0.25) | .13 |
| **Lifestyle** | |  |  |  |  |  |  |  |  |
| Physical activity (hours) | | 0.06 (0.02) | **.02** | 0.01 (0.02) | .67 | 0.03 (0.01) | **.03** | 0.02 (0.01) | **.03** |
| Body mass index (kg/m^2^) | | -0.04 (0.09) | .65 | -0.04 (0.07) | .53 | -0.05 (0.05) | .26 | 0.06 (0.02) | **.003** |
| Smoking categories (ref=never) | |  |  |  |  |  |  |  |  |
|  | Former smoker | 2.13 (0.72) | **.003** | 0.95 (0.43) | **.03** | 1.42 (0.49) | **.004** | -0.08 (0.22) | .70 |
|  | Current smoker | 3.33 (1.43) | **.02** | 1.97 (1.06) | .06 | 0.83 (0.77) | .28 | 0.59 (0.44) | .18 |
| **Clinical factors** | |  |  |  |  |  |  |  |  |
| Months since diagnosis ^a^ | | -0.05 (0.02) | **.002** | -0.01 (0.01) | .42 | -0.03 (0.01) | **.003** | -0.01 (0.01) | **.04** |
| Tumor location (ref=colon cancer) | | 0.29 (1.22) | .82 | -0.11 (1.10) | .92 | 0.42 (0.69) | .55 | 0.03 (0.27) | .93 |
| Tumor staging (ref=stage I) | |  |  |  |  |  |  |  |  |
|  | Stage II | -0.19 (1.13) | .86 | -0.05 (0.97) | .96 | -0.17 (0.55) | .76 | 0.07 (0.27) | .80 |
|  | Stage III or IV | -0.13 (1.07) | .90 | -1.07 (0.78) | .17 | 1.02 (0.65) | .12 | -0.08 (0.24) | .75 |
| Chemotherapy (ref=no) | | -0.80 (1.04) | .44 | -0.13 (0.74) | .86 | -1.19 (0.61) | **.05** | 0.52 (0.28) | .06 |
| Radiotherapy (ref=no) | | -0.04 (1.53) | .98 | 0.20 (1.26) | .87 | 0.09 (0.88) | .92 | -0.34 (0.34) | .32 |
| Stoma placement (ref=no) | | -0.92 (1.11) | .41 | -0.84 (0.54) | .12 | -0.31 (0.58) | .59 | 0.34 (0.33) | .31 |
| Number of comorbidities (ref=none) | |  |  |  |  |  |  |  |  |
|  | 1 | -0.65 (0.60) | .28 | -0.28 (0.42) | .51 | -0.46 (0.37) | .21 | 0.00 (0.19) | .99 |
|  | ≥2 | -1.24 (0.66) | .06 | -0.77 (0.47) | .10 | -0.67 (0.42) | .11 | 0.04 (0.21) | .86 |
| **Footnotes**: a) Months since diagnosis was entered into the models as a measure of time | | | | | | | | | |
